# Mokko Lactone Alleviates Doxorubicin-Induced Cardiotoxicity in Rats via Antioxidant, Anti-Inflammatory, and Antiapoptotic Activities

**DOI:** 10.3390/nu14040733

**Published:** 2022-02-09

**Authors:** Alaa Sirwi, Rasheed A. Shaik, Abdulmohsin J. Alamoudi, Basma G. Eid, Mahmoud A. Elfaky, Sabrin R. M. Ibrahim, Gamal A. Mohamed, Hossam M. Abdallah, Ashraf B. Abdel-Naim

**Affiliations:** 1 Department of Natural Products, Faculty of Pharmacy, King Abdulaziz University, Jeddah 21589, Saudi Arabia; asirwi@kau.edu.sa (A.S.); melfaky@kau.edu.sa (M.A.E.); gahussein@kau.edu.sa (G.A.M.); hmafifi@kau.edu.sa (H.M.A.); 2 Department of Pharmacology and Toxicology, Faculty of Pharmacy, King Abdulaziz University, Jeddah 21589, Saudi Arabia; rashaikh1@kau.edu.sa (R.A.S.); ajmalamoudi@kau.edu.sa (A.J.A.); beid@kau.edu.sa (B.G.E.); 3 Centre for Artificial Intelligence in Precision Medicines, King Abdulaziz University, Jeddah 21589, Saudi Arabia; 4 Department of Chemistry, Preparatory Year Program, Batterjee Medical College, Jeddah 21442, Saudi Arabia; sabrin.ibrahim@bmc.edu.sa; 5 Department of Pharmacognosy, Faculty of Pharmacy, Assiut University, Assiut 71526, Egypt; 6 Department of Pharmacognosy, Faculty of Pharmacy, Cairo University, Cairo 11562, Egypt

**Keywords:** doxorubicin, mokko lactone, heart

## Abstract

Doxorubicin (DOX), a commonly utilized anthracycline antibiotic, suffers deleterious side effects such as cardiotoxicity. Mokko lactone (ML) is a naturally occurring guainolide sesquiterpene with established antioxidant and anti-inflammatory actions. This study aimed at investigating the protective effects of ML in a DOX-induced cardiotoxicity model in rats. Our results indicated that ML exerted protection against cardiotoxicity induced by DOX as indicated by ameliorating the rise in serum troponin and creatine kinase-MB levels and lactate dehydrogenase activity. Histological assessment showed that ML provided protection against pathological alterations in heart architecture. Furthermore, treatment with ML significantly ameliorated DOX-induced accumulation of malondialdehyde and protein carbonyl, depletion of glutathione, and exhaustion of superoxide dismutase and catalase. ML’s antioxidant effects were accompanied by increased nuclear translocation of NF-E2-related factor 2 (Nrf2) and heme oxygenase-1 (HO-1) expression. Moreover, ML exhibited significant anti-inflammatory activities as evidenced by lowered nuclear factor κB, interleukin-6, and tumor necrosis factor-α expression. ML also caused significant antiapoptotic actions manifested by modulation in mRNA expression of Bax, Bcl-2, and caspase-3. This suggests that ML prevents heart injury induced by DOX via its antioxidant, anti-inflammatory, and antiapoptotic activities.

## 1. Introduction

Doxorubicin (DOX) is a commonly utilized anthracycline antibiotic for treating several types of cancer, including breast cancer and lymphomas [1]. However, its clinical applications are relatively restricted due to its detrimental side effects that include cardiotoxicity [2]. It was reported that the incidence of acute DOX cardiotoxicity is around 11%, which can be characterized on the electrocardiogram by decreased amplitude of QRS complexes and nonspecific ST changes [3,4]. This toxic damage to the cardiomyocytes induced by DOX can lead to the development of tachycardia, arrhythmia, pericarditis, myocarditis, left ventricular function transient depression, late-onset refractory cardiomyopathy, and, eventually, congestive heart failure [5,6]. Unfortunately, the development of congestive heart failure with DOX therapy indicates a poor prognosis of cancer patients, as it is associated with nearly 50% mortality in 1 year [7]. DOX cardiomyopathy is characterized histopathologically by patchy interstitial fibrosis and myocyte vacuolar degeneration [8,9]. DOX cardiotoxicity is usually accompanied by raised troponin, creatine kinase isoenzyme MB (CK-MB), and lactate dehydrogenase (LDH) levels in the serum [10]. DOX can induce dose-related cardiomyopathy through multiple mechanisms that involve increased oxidative stress, as shown by the increased cardiac generation of oxygen free radicals and lipid peroxidation products [11,12,13].

Since DOX is a useful chemotherapy drug in cancer, different approaches have been adopted to alleviate its toxic side effects that include dosage optimization, combination therapy, and the development of analogs. However, no satisfactory results have been achieved out of these ongoing efforts [14]. Therefore, cardioprotection during DOX treatment is required to reduce the incidence of DOX-induced heart damage; hence, it is necessary to discover new drugs that can be utilized as cardioprotective agents with DOX therapy. In this regard, natural products remain an attractive source of bioactive lead compounds that can tackle this problem [15]. With regards to disease treatment and prevention, natural products are still considered one of the best sources of novel bioactive molecules. The potential of research efforts in this field is highlighted by the fact that 16% of the US-FDA drug approvals in 2018 were for new drugs classified as natural products [16]. Phytoconstituents are considered a source of bioactive compounds that could lead to new drugs. In this regard, rhizomes of *Costus speciosus* (*Zingiberaceae*) are traditionally utilized in Indian folk medicine for their anti-inflammatory, antispasmodic, hepatoprotective, antidiabetic, antihyperlipidemic, antimicrobial, and anthelmintic activities. Moreover, it was shown that the rhizomes of this plant exert cardioprotective effects against oxidative stress in atherosclerotic [17]. The rhizomes are rich in different phytoconstituents, mainly guaianolides sesquiterpene lactones [18,19]. Guaianolides have several reported pharmacological effects that include antioxidant, anti-inflammatory, and antimicrobial activities [20]. Mokko lactone (ML, dihydrodehydrocostus lactone) is a major guaianolide in *C. speciosus* that possesses notable anti-inflammatory action, as it has been shown to significantly reduce the release of TNF-α and IL-6 from stimulated human peripheral blood mononuclear cells [21]. ML has also demonstrated significant antioxidant and hepatoprotective effects in rats challenged with DOX [22]. Thus, this work aimed at examining the possible protection offered by ML, extracted from the rhizomes of *C. speciosus*, against acute cardiotoxicity induced by DOX in rats.

## 2. Materials and Methods

### 2.1. Chemicals

ML (purity > 98%) was isolated from *Costus speciosus* rhizomes extract (Supplementary Materials). DOX HCl was obtained from Sigma-Aldrich (St. Louis, MO, USA). Remaining chemicals conformed with the highest available commercial purity.

### 2.2. Animals

Twenty-four Wistar rats (males, 200–230 g) were purchased from the animal facility, Faculty of Pharmacy, King Abdulaziz University. Animals were kept on a 12-h light/dark cycle at ambient temperature (22 ± 3 °C) with humidity (60–70%), with access to food and water. Research Ethics Committee, Faculty of Pharmacy, King Abdulaziz University approved the experimental protocol (Reference # PH-1443-13).

### 2.3. Toxicity Study

Acute oral toxicity of ML was assessed according to OECD guideline number 423. Briefly, rats were given a single oral dose of 2000 mg/kg. After treatment, animals were individually observed at least one time during the first hour and regularly for the upcoming 24 h, with particular attention during the first 4 h. Since all rats survived, the experiment was repeated using three additional male rats.

### 2.4. Experimental Protocol

Animals were placed in groups of four in a random fashion (*n* = 6): controls, the DOX group, the first treatment group was given 15 mg/kg ML and DOX while the second treatment group was given 30 mg/kg ML and DOX. The doses of ML were chosen after carrying out a pilot study and were consistent with those in the literature [22]. Control and DOX groups were given 0.5% carboxymethyl cellulose (CMC) orally one time daily for 10 days consecutively. ML was suspended in CMC and was administered to both treatment groups orally for 10 days at the mentioned doses. On the tenth day, controls were given an intraperitoneal (IP) injection of 0.9% saline, 60 min after ML administration. Similarly, on the tenth day and 60 min after ML the remaining groups were administered an IP dose of 15 mg/kg DOX dissolved in 0.9% saline. Volume used for dosing all animals was 10 mL/kg. Twenty-four hours post last injection, animals were given 50 mg/kg ketamine and 5 mg/kg xylazine IP for anesthesia. Electrocardiogram (ECG) measurements were then performed on the animals. The retroorbital plexus was used for collecting blood samples. Blood was kept for 15 min and then centrifuged for 10 min at 3000 RPM and 4 °C to obtain serum. Decapitation was performed, and hearts were dissected out, gently rinsed with saline (ice-cooled), and blotted between filter paper. Part of the heart was placed in 10% formalin for histopathological and immunohistochemical studies. Remaining sections of the hearts were placed in RNAProtect Tissue Reagent (Cat. No. 76106, Qiagen, MD, USA). The remaining parts were flash-frozen with liquid nitrogen and held together with serum at −80 °C for analysis.

### 2.5. Electrocardiography

At the end of the treatment protocol, animals were given a combination of ketamine (100 mg/kg; i.p.) + xylazine (10 mg/kg; i.p.) for anesthesia. During electrocardiography, rectal temperatures were kept at 37.5 °C by a thermostatically controlled heating blanket. In all animals, 10 min after anesthesia, three needle electrodes were placed below the skin of the animals. Electrodes were placed in the right hind and front limbs and the left hind limb. A PowerLab, model 8/35 (ADInstruments, Sydney, Australia), was used to record the ECG. ECG parameters were recorded. The changes in duration of P wave (ms), QRS complex (ms), QRS amplitude (µV), QT interval (ms), PR interval (ms), RR interval (ms), and amplitude of ST segment (µV) were determined.

### 2.6. Biochemical Assays and Measurements of Cardiac Enzymes

Serum was collected from blood samples by centrifugation for 10 min at 3000 rpm and placed in Eppendorf tubes for biochemical analysis of creatine kinase myocardial band (CK-MB), cardiac troponin levels, and lactate dehydrogenase (LDH), and were assessed using colorimetric kits (SEA479Ra, SEA478Ra, and SEB370Ra, Cloud-Clone, Houston, TX, USA, respectively).

### 2.7. Histopathological Study

Heart tissues were fixed in 10% neutral formalin, and then paraffinization was performed. Tissues were cut into slices (5 µm). Hematoxylin and eosin (H&E) was used to stain the sections, which were then photographed using light microscopy (Nikon Eclipse TE2000-U, Nikon, Tokyo, Japan). This examination was carried out by a pathologist in a blind manner.

### 2.8. Assessment of Oxidative and Inflammatory Markers

Homogenization of heart tissues was carried out in a 10-fold volume of ice-cooled phosphate-buffered saline (PBS) (ice-cooled, pH 7.4). Following centrifugation at 10,000× *g* for 20 min and 4 °C, the supernatant was then collected for analysis of oxidative and inflammatory markers. ELISA kits were used in the assessment of the hearts’ content of malondialdehyde (MDA), reduced glutathione (GSH), enzymatic activities of superoxide dismutase (SOD), catalase (CAT) (Cat. No. MD 2529, GR 2511, SD 2521, and CA 2517, Biodiagnostic, Giza, Egypt, respectively), and protein carbonyl, interlukin-6 (IL-6), and tumor necrosis factor α (TNF-α) (Cat. No. ab238536, ab234570, and ab100785, Abcam, Cambridge, UK, respectively).

Nuclear fractions of tissue homogenates were obtained using NE-PER nuclear and cytoplasmic extraction kit (Cat. No. 78833, Thermo Fisher Scientific, Waltham, MA, USA). Protein content of the nuclear extracts was determined, and a volume equivalent to 80 μg was employed in the assessment of the DNA-binding activity of NF-kB p65 using NF-κB p65 ELISA Kit (Cat. No. ab133112, Abcam, Cambridge, UK). Results are expressed as fold change of control.

### 2.9. Tissue Staining for Immunohistochemistry

Tissue sections were deparaffinized, and then ethanol serial dilutions were employed for tissue rehydration before boiling in 0.1 M citrate buffer (pH 6.0) for 10 min. A 2-hour incubation period in 5% bovine serum albumin (BSA) in tris buffered saline (TBS) was subsequently followed. Primary antibodies were then used in the tissue incubation for 12 h at 4 °C, namely: TNFα (Cat. No. ab220210, Abcam^®^, Cambridge, UK), IL-6, (Cat. No. ab9324, Abcam^®^, Cambridge, UK), NFκB (Cat. No. sc-8414, Santa Cruz, TX, USA), and Nrf2 (Cat. No. MBS9608128, MyBioSource, San Diego, CA, USA). After tissue flushing using TBS, another incubation was carried out in either antimouse or antirabbit biotinylated secondary antibody based on the primary antibody reactivity (Cell & Tissue Staining Kit, Cat. No. CTS002, CTS006, R&D systems, Minneapolis, MN, USA). Quantification was performed with an image analysis software (Image J, 1.8.0, NIH, Bethesda, MD, USA).

### 2.10. Quantitative Real-Time Polymerase Chain Reaction (PCR)

TRIzol was used for isolation of total RNA from the tissues of the heart. A260/A280 ratio was employed in assessing RNA purity. Samples with a ratio greater than 1.7 were included in the synthesis of cDNA. Omniscript RT kit (Cat. No. 205113, Qiagen, MD, USA) was used for first-strand cDNA synthesis. A SYBR Green Master Mix (Cat. No. 180830, Qiagen, MD, USA) with forward and reverse primers was used for quantification of mRNA using qPCR. The following sequences represented the forward primers for Nrf2, HO-1, Bax, Bcl-2, caspase-3, and β-actin: 5′TTTGTAGATGACCATGAGTCGC,5′TCTGCAGGGGAGAATCTTGC, 5′CCTGAGCTGACCTTGGAGCA, 5′TGATAACCGGGAGATCGTGA, 5′CTCGGTCTGGTACAGATGTCGATG, and 5′TCCGTCGCCGGTCCACACCC, respectively. The following sequences represented the reverse primers for Nrf2, HO-1, Bax, Bcl-2, caspase-3 and β-actin: 5′TGTCCTGCTGTATGCTGCTT, 5′TTGGTGACGGAAATGTGCCA, 5′GGTGGTTGCCCTTTTCTACT, 5′AAAGCACATCCAATAAAAAGC, 5′GGTTAACCCGGGTAAGAATGTGCA, and 5′TCACCAACTGGGACGATATG, respectively. The primers sequences were taken from references that have been already validated in our laboratory [22]. Data were analyzed by the ΔΔCT method, and β-actin was used for normalization [23].

### 2.11. Statistics

Data are represented as means ± SD. One-way ANOVA followed by Tukey’s multiple comparison test was used for assessing results. GraphPad Prism (Prism 8.1, GraphPad Software, Inc., La Jolla, CA, USA) was used for all analyses. *p* < 0.05 was considered significant.

## 3. Results

### 3.1. Assessment of Acute Toxicity of ML

At 24 h of oral ML dose of 2000 mg/kg to rats, no deaths were observed in three tested male animals. A further study was carried out in three male animals utilizing the same dose, which, similarly, resulted in no deaths after 24 h.

### 3.2. Assessment of Heart Electrical Activities

**Figure 1:**
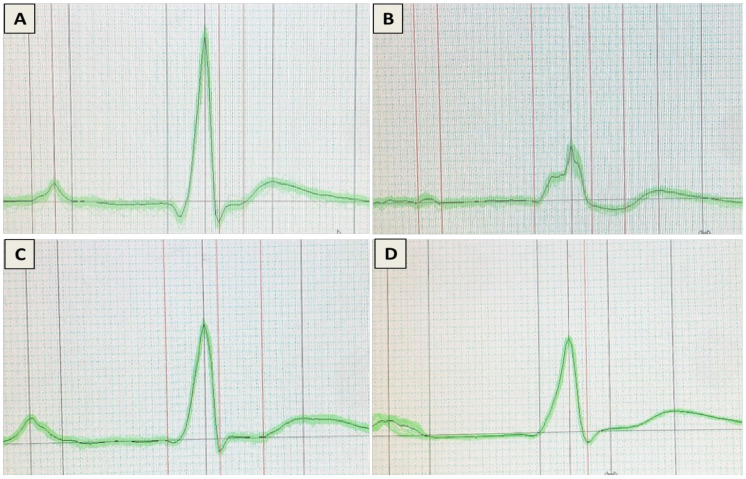
Effect of ML on DOX-induced on ECG patterns: (**A**) control group; (**B**) DOX group; (**C**) DOX + ML (15 mg/kg); and (**D**) DOX + ML (30 mg/kg), DOX = Doxorubicin, ML = Mokko Lactone.

Electrocardiographic patterns (P wave duration, QRS complex duration, QRS amplitude, QT interval, PR interval duration, RR interval duration, ST segment amplitude) of the control and experimental groups are displayed in Table 1 and Figure 1. The values of ECG indices are given in Table 1. Control rats had normal ECG findings, while the DOX-challenged rats demonstrated a markedly lowered P wave and QRS complex. Furthermore, DOX injection induced a significant increase in the QT, PR, and RR intervals and ST segment relative to controls. However, these pathological changes in P wave magnitude and QRS amplitude and complexes were prevented by ML in a dose-related manner. ML also resulted in a significant restoration of the QT, PR, and RR intervals and ST segment.

### 3.3. Histopathological Assessment

**Figure 2:**
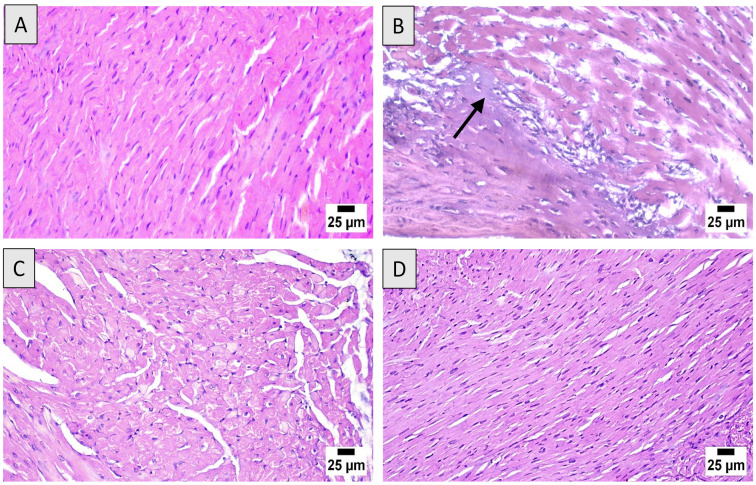
Effect of ML on DOX-induced histopathological changes on cardiac tissues: (**A**) control group demonstrating normal architecture of the heart tissues; (**B**) DOX group exhibiting mononuclear inflammatory cells infiltration and widespread necrosis of cardiac tissues (black arrow); (**C**) DOX + ML (15 mg/kg) treated group showing limited inflammatory areas and degenerated cardiomyocytes; (**D**) DOX + ML (30 mg/kg) treated group with restoration of a nearly normal cardiac histology. DOX = Doxorubicin, ML = Mokko Lactone.

Microscopic examination of heart sections from the control group revealed a normal histological structure of cardiac architecture (Figure 2A). However, the DOX group revealed marked cardiotoxicity that was characterized by scattered degeneration of cardiac myofibers, mononuclear inflammatory cells infiltration, myocarditis, and extensive cytoplasmic vacuolization (Figure 2B). Sections from DOX + ML (15 mg/kg) showed moderate enhancement of the cardiac histology with fewer inflammatory areas and degenerated cardiomyocytes (Figure 2C). Rats given DOX + ML (30 mg/kg) showed the highest protection that revealed an apparently normal structure in most examined sections (Figure 2D).

Data in Table 2 show semi-quantitatively the cardiac injury induced by DOX. The pathological alterations included severe disruption of cardiac muscles architecture, interstitial edema, inflammatory cellular infiltrate, apoptosis, and necrosis, as evidenced by nuclear pyknosis, karyorrhexis, and/or karyolysis. Both doses of ML obviously ameliorated such deleterious effects to the borderline score in cardiac myocyte death in animals treated with the 30 mg/kg ML.

### 3.4. Effect of ML on Serum Cardiac Markers

**Figure 3:**
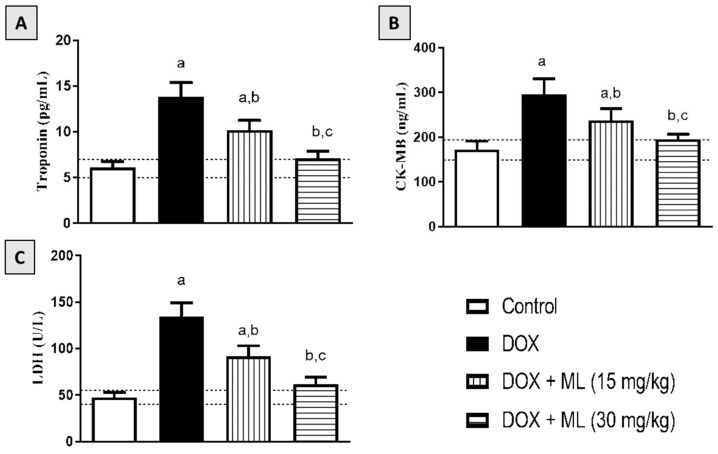
Effect of ML on serum cardiac markers in DOX-treated rats: (**A**) serum troponin level; (**B**) serum CK-MB level; (**C**) serum LDH activity. Background dotted lines represent range of corresponding control values. Data are displayed as mean ± SD (*n* = 6). DOX = Doxorubicin, ML = Mokko Lactone. a, significantly different than control (*p* < 0.05); b, significantly different than DOX (*p* < 0.05); c, significantly different than DOX + ML (15 mg/kg) (*p* < 0.05).

The protective activity of ML against DOX-induced heart injury was confirmed based on the levels of serum markers of cardiotoxicity, namely, troponin, CK-MB, and LDH. As can be observed in Figure 3A,B, rats challenged with DOX showed raised troponin and CK-MB levels in the serum compared with control rats. However, treatment with ML significantly ameliorated and prevented this DOX-induced increase in both markers at 15 mg/kg and 30 mg/kg, respectively. The data in Figure 3C reveal that prior treatment with ML at 15 mg/kg to DOX-challenged rats significantly ameliorated the increase in serum LDH activity by approximately 32%; this increase was significantly inhibited at an ML dose level of 30 mg/kg.

### 3.5. Effect of ML on Cardiac Oxidative Stress

**Figure 4:**
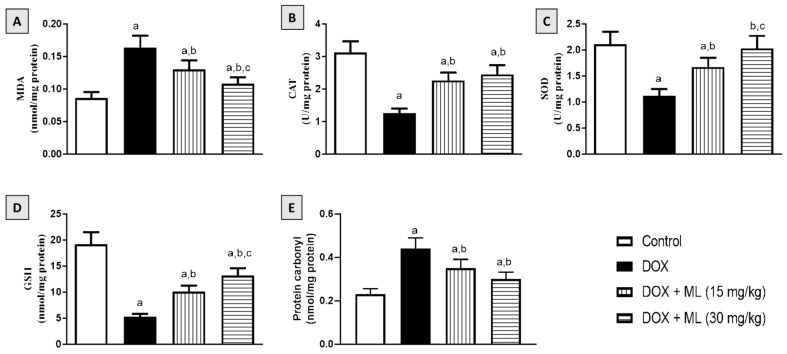
Effect of ML on oxidative status of on DOX-induced cardiotoxicity in rats: (**A**) cardiac MDA content; (**B**) cardiac CAT activity; (**C**) cardiac SOD activity; (**D**) cardiac GSH; and (**E**) cardiac protein carbonyl content. Data are displayed as mean ± SD (*n* = 6). DOX = Doxorubicin, ML = Mokko Lactone. a, significantly different compared with control (*p* < 0.05); b, significantly different compared with DOX (*p* < 0.05); c, significantly different compared with DOX + ML (15 mg/kg) (*p* < 0.05).

The antioxidant activity of ML was also examined in rats following acute DOX exposure. Figure 4A shows that DOX resulted in increased oxidative stress, as shown by the increased contents of MDA. Nonetheless, treatment with ML significantly ameliorated the increase in MDA by around 20% and 33% at 15 mg/kg and 30 mg/kg, respectively. It can also be observed in Figure 4B–D that the DOX challenge led to significant GSH depletion and CAT and SOD exhaustion. However, treatment with ML at both doses tested significantly attenuated the depletion of GSH, increasing the values by 92.3% and 151.9% relative to the DOX group, respectively. ML also significantly ameliorated the exhaustion of CAT and SOD associated with DOX-induced oxidative stress at both doses tested in a concentration-related manner. In addition, ML significantly ameliorated the increase in protein carbonyl associated with DOX-induced oxidative stress at both doses tested in a dose-related manner (Figure 4E).

### 3.6. Effect of ML on Nrf2 and HO-1 Expression

**Figure 5:**
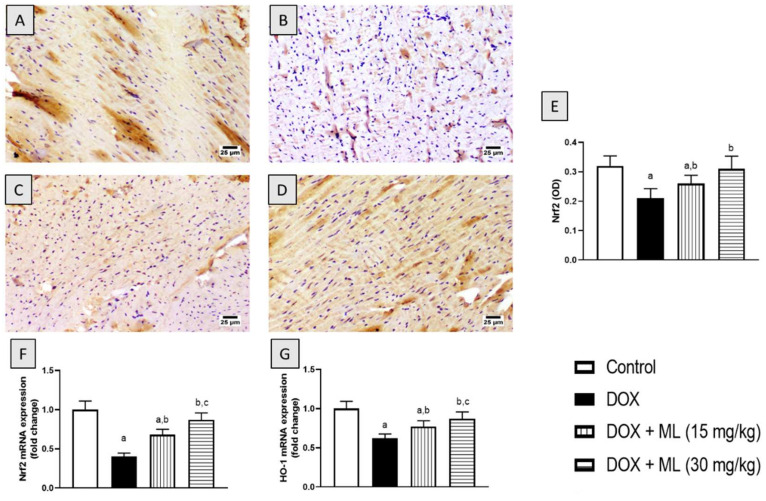
Effect of ML on Nrf2 expression as determined immunohistochemically (**A**–**E**) and mRNA expression of Nrf2 (**F**) and HO-1 (**G**) in cardiac tissues of DOX-treated rats. Data shown as bar charts are mean ± SD (*n* = 6). DOX = Doxorubicin, ML = Mokko Lactone. a, b, or c: statistically different from control, DOX, or DOX + ML (15 mg/kg), respectively (*p* < 0.05).

To confirm the antioxidant activity and to assess the anti-inflammatory potential of ML, the cardiac expression of Nrf2 was examined following ML and DOX treatments. As it can be observed in Figure 5A–E, DOX caused a marked lowering of Nrf2 expression in comparison to the control value. Interestingly, treatment with ML not only ameliorated this decrease in Nrf2 at 15 mg/kg by approximately 50% but also significantly prevented it at 30 mg/kg. In addition, these data were confirmed by assessing Nrf2 and HO-1 mRNA expression, which were significantly downregulated by DOX challenge. However, both doses of ML ameliorated such effects and significantly inhibited the decrease in mRNA expression of Nrf2 and HO-1 (Figure 5F,G).

### 3.7. Effect of ML on Expression of Heart Inflammatory Markers

**Figure 6:**
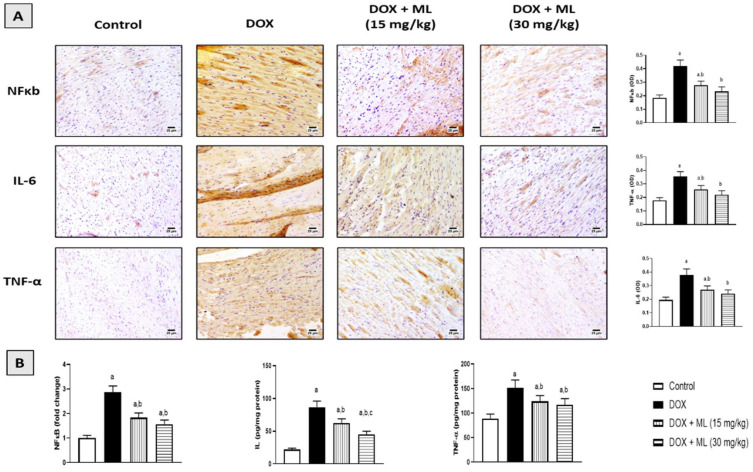
Effect of ML on NFκB, IL-6, and TNF-α, as determined by immunohistochemistry (**A**) or ELISA (**B**) in cardiac tissues of DOX-treated rats. Data in bar charts are mean ± SD (*n* = 6). DOX = Doxorubicin, ML = Mokko Lactone. a, b or c: statistically significant compared with control, DOX, or DOX + ML (15 mg/kg), respectively (*p* < 0.05).

The inflammatory status of cardiac tissues was examined immunohistochemically in DOX-challenged rats following ML treatment. DOX challenge significantly induced the expression of NF-κb, while ML treatment significantly attenuated the increased expression of NF-κb at 15 mg/kg and 30 mg/kg by 33.8% and 44.7%, respectively. Moreover, the expression of IL-6 and TNF-α was also increased with DOX and this was significantly ameliorated by ML at 15 mg/kg by 28.9% and 26.7%, respectively. ML at 30 mg/kg resulted in an even further decrease in the expression of IL-6 by 36.3% and TNF-α by 38.0% compared with DOX alone (Figure 6A). These data were confirmed using the ELISA technique that indicated the ability of DOX to activate NF-κb and the cardiac content of IL-6 and TNF-α. The same protective actions of both doses of ML were observed. ML treatment was associated with the significant amelioration of NF-κb DNA-binding activity and prevented the rise in IL-6 and TNF-α content (Figure 6B).

### 3.8. Effect of ML on Bax, Bcl-2, and Caspase-3 mRNA Expression

**Figure 7:**
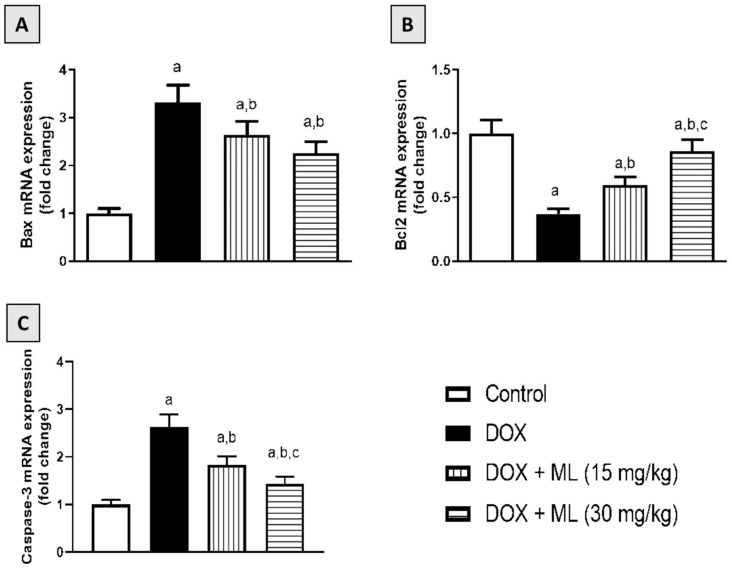
Effect of ML on cardiac mRNA expression of Bax (**A**), Bcl-2 (**B**), and caspase-3 (**C**) in DOX-treated rats. Data shown in bar charts are mean ± SD (*n* = 6). DOX = Doxorubicin, ML = Mokko Lactone. a, b or c: statistically significant compared with control, DOX, or DOX + ML (15 mg/kg), respectively (*p* < 0.05).

ML’s antiapoptotic effects were examined based on the Bax, Bcl2, and caspase-3 mRNA expression in cardiac tissues of rats who received a single DOX injection. As demonstrated in Figure 7A, DOX resulted in a significant increase in the mRNA expression of the proapoptotic regulator Bax. However, ML markedly ameliorated this rise by 21.2% and 33.3% at 15 mg/kg and 30 mg/kg, respectively. Regarding the antiapoptotic Bcl-2, DOX significantly decreased Bcl-2 mRNA expression while ML significantly ameliorated this change, as it enhanced its values by more than one- and two-fold at 15 mg/kg and 30 mg/kg, respectively (Figure 7B). In addition, DOX showed significant apoptotic activities, as evidenced by enhanced mRNA expression of caspase-3. ML markedly ameliorated this rise by approximately 18% and 25% at 15 mg/kg and 30 mg/kg, respectively (Figure 7C).

## 4. Discussion

Cardiotoxicity associated with doxorubicin is acute, occurring within 2 days of its administration, and acute cardiotoxicity occurs in approximately 11% of cases [24]. However, DOX is frequently utilized in anticancer treatment protocols because of the high rates of complete remission associated with this agent compared with many other drugs [25]. DOX-induced cardiotoxicity mechanism is complex and multifaceted and involves the induction of oxidative stress [26]. ML is a sesquiterpene lactone obtained from the rhizomes of *Costus speciosus,* which has been shown to have significant antioxidant and anti-inflammatory properties [20,27]. Hence, we aimed at investigating the possible protective actions of ML due to DOX-induced cardiotoxicity in rats.

Oral LD50 examination of ML in rats indicated almost no toxicity of the compound. On the experiments of electrical potential generated by the heart, our data demonstrated that DOX challenge in rats induced acute cardiotoxicity, as indicated by the alterations in ECG indices [28]. The administration of ML significantly ameliorated these changes in P wave, QRS complex, QT/RR intervals, and ST segments at 15 mg/kg and prevented them at 30 mg/kg. Moreover, ML inhibited cardiac myopathy, as indicated by the histopathological examination of heart tissues, as ML treatment was associated with relatively preserved cardiomyocytes and almost normal cardiac architecture, indicating a protective activity of ML. According to these functional and histological parameters, it appears that ML can prevent DOX-induced cardiac toxicity in a dose-related manner. Cardiac dysfunction associated with DOX occurs via varied mechanisms that primarily include the induction of oxidative stress leading to severe cellular injury [29]. The results obtained in the current work indicate significant antioxidant activity of ML, as evidenced by the amelioration of pathological changes induced by DOX to the oxidative stress markers MDA, CAT, SOD, GSH, and carbonyl in cardiac tissues. These antioxidant effects could be attributed to the α-methylene-γ-lactone moiety in the chemical structure of ML that confers the ability to interact with the cysteine sulfhydryl groups of many cellular peptides and proteins [30]. These results are consistent with the reported antioxidant activity of ML in DOX-induced hepatotoxicity [22]. Hence, these results indicate that ML decreases oxidative stress and cellular injury in cardiac tissues.

Nrf2 expression analysis further confirmed the antioxidant activity of ML against DOX-induced oxidative stress in cardiac tissues. DOX challenge significantly reduced the cardiac expression of Nrf2. However, treatment with ML significantly prevented this decrease in Nrf2 induced by the DOX challenge. Nrf2 is known to be heavily involved in mediating cellular resistance to oxidative stress in DOX-induced cardiotoxicity [31,32,33]. Hence, the antioxidant action associated with ML administration be positively regulated by Nrf2 expressed in cardiomyocytes. Furthermore, Nrf2 is also known to reduce inflammatory injury via the regulation of inflammatory cytokines and antioxidant enzymes [34].

The finding of the current study showed increased expression of proinflammatory mediators and cytokines with DOX challenge, while this increase was prevented with ML treatment in a dose-related manner. It has been suggested that cardiac inflammation plays a significant role in DOX-related cardiotoxicity. Inhibiting inflammation has even been shown to facilitate recovering heart dysfunction following DOX administration [35]. These findings are in harmony with other findings in the literature demonstrating the significant anti-inflammatory activity of ML, as evidenced by the reduced release of proinflammatory cytokines, including IL-6 and TNF-α, from activated human peripheral blood mononuclear cells [21]. Thus, this observed anti-inflammatory activity could contribute to the protective effects of ML against cardiotoxicity induced by DOX.

It is documented that DOX cardiotoxicity was likened to the increased apoptotic potential of cardiomyocytes [36]. In this regard, ML was found in the current study to protect against the apoptosis of cardiac tissues in rats who received DOX. Interestingly, the observed antiapoptotic changes in expression of Bax, Bcl2, and caspase-3 caused by ML may be due to Nrf2 dependent mechanisms. It is known that Nrf2 enhances resistance to apoptotic stimuli by upregulating the antiapoptotic protein Bcl2 [37]. It has also been shown that a structurally related compound, costunolide, protects against apoptosis mediated by oxidative stress in a defense mechanism that is dependent on Nrf2 expression and involves antiapoptotic changes in the expression of Bax and Bcl2 [38,39,40]. Taken together, the generated findings in the current study highlight the importance of the antioxidant, anti-inflammatory, and antiapoptotic activities of ML in mediating resistance to the acute cardiotoxic effects of DOX in rats.

## Figures and Tables

**Table 1:** Effect of ML on DOX-induced alterations in electrocardiographic (ECG) indices.

	Control	DOX	DOX + ML (15 mg/kg)	DOX + ML (30 mg/kg)
P wave (duration, ms)	30 ± 1 (29–30)	22 ± 5 ^a^ (17–27)	26 ± 1 (25–27)	34± 1 ^b^ (33–35)
QRS complex (duration, ms)	63 ± 2 (61–65)	30 ± 1 ^a^ (29–31)	40 ± 2 (38–42)	65 ± 1 ^b^ (64–66)
QRS amplitude (µV)	82 ± 2 (80–84)	140 ± 10 ^a^ (130–150)	60 ± 2 ^b^ (58–62)	80± 1 ^b^ (79–81)
QT interval (duration, ms)	50 ± 1 (49–51)	72 ± 3 ^a^ (69–75)	60 ± 1 (59–61)	54± 1 ^b^ (53–55)
PR interval (duration, ms)	20 ± 1 (19–21)	25 ± 1 ^a^ (24–26)	22 ± 1 (21–23)	19 ± 1 ^b^ (18–20)
RR interval (duration, ms)	150 ± 1 (149–151)	260 ± 1 ^a^ (259–261)	220 ± 3 (217–223)	130 ± 3 ^b^ (127–133)
ST segment amplitude (µV)	51 ± 3 (48–54)	180 ± 3 ^a^ (177–183)	100 ± 3 (97–103)	55± 1 ^b^ (54–56)

Results are displayed as mean ± SD (*n* = 6) and range of values in each group is shown between brackets; DOX = Doxorubicin, ML = Mokko Lactone, ^a^ significantly different from control (*p* < 0.05); ^b^ significantly different from DOX (*p* < 0.05).

**Table 2:** Histopathological semi-quantitative scoring showing the effects of ML on DOX-induced severity of histopathologic lesions in DOX-treated rats.

	Control	DOX	DOX + ML (15 mg/kg)	DOX + ML (30 mg/kg)
Disruption of cardiac muscles	-	+++	++	+
Interstitial edema	-	+++	++	+
Inflammatory cellular infiltrate	-	+++	++	+
Apoptosis	-	++	+	±
Necrosis (nuclear pyknosis, karyolysis, karyorrhexis)	-	++	+	±

Score values are obtained from tissue sections of six animals of each group, five fields/section (X 100): scores of -, normal; ±, borderline; +, mild; ++, moderate; +++, severe. DOX = Doxorubicin, ML = Mokko Lactone.

## Data Availability

Data are contained within the article and the Supplementary Materials.

## Supplementary Materials

The following supporting information can be downloaded at: https://www.mdpi.com/article/10.3390/nu14040733/s1, Figure S1: Chemical structure of mokko lactone, Figure S2: ESIMS of mokko lactone, Figure S3: 1H NMR spectrum of mokko lactone in CDCl3 (600 MHz), Figure S4: 13C NMR spectrum of mokko lactone in CDCl3 (150 MHz).
