# Supplementary material for: Mokko Lactone Alleviates Doxorubicin-Induced Cardiotoxicity in Rats via Antioxidant, Anti-Inflammatory, and Antiapoptotic Activities
Source: Nutrients. 2022 Feb 9;14(4):733. doi: 10.3390/nu14040733 (PMC8880813; doi:10.3390/nu14040733)
Supplement: Supplementary file 1 [file nutrients-14-00733-s001.zip › nutrients-1552405-supplementary.pdf]

Mokko Lactone Alleviates Doxorubicin-Induced Cardiotoxicity in Rats via Antioxidant, Anti-Inflammatory, and Antiapoptotic Activities

Alaa Sirwi [1], Rasheed A . Shaik [2], Abdulmohsin J. Alamoudi [2], Basma G. Eid [2], Mahmoud A. Elfaky [1,3], Sabrin R. M. Ibrahim [4,5], Gamal A. Mohamed [1], Hossam M. Abdallah [1,6] and Ashraf B. Abdel-Naim [2,*]

[1] Department of Natural Products, Faculty of Pharmacy, King Abdulaziz University, Jeddah 21589, Saudi Arabia

[2] Department of Pharmacology and Toxicology, Faculty of Pharmacy, King Abdulaziz University, Jeddah 21589, Saudi Arabia

[3] Centre for Artificial intelligence in Precision Medicines, King Abdulaziz University, Jeddah 21589, Saudi Arabia

[4] Department of Chemistry, Preparatory Year Program, Batterjee Medical College, Jeddah 21442, Saudi Arabia

[5] Department of Pharmacognosy, Faculty of Pharmacy, Assiut University, Assiut 71526, Egypt

[6] Department of Pharmacognosy, Faculty of Pharmacy, Cairo University, Cairo 11562, Egypt

[*] Correspondence: aaabdulalrahman1@kau.edu.sa; Tel.: +966-55-6814781

General experimental procedures

ESIMS spectrum was measured utilizing a LCQ DECA mass spectrometer. BRUKER AVANCE 600 was utilized for NMR spectra measuring. Chromatographic separation was performed on SiO_2 60 (0.04-0.063 mm) and RP-18 (0.04–0.063 mm). TLC analysis was performed on pre-coated TLC plates with SiO_2 60 F_{254} (0.2 mm). The compound was detected by UV absorption (λ_{max} 255 and 366 nm), followed by spraying with *p*-anisaldehyde:H_2SO_4 and heating at 110°C.

Plant material

In March 2020, *C. speciosus* rhizomes were purchased from an authorized local market in Jeddah governorate, KSA. The plant's authentication was proved by Dr. Emad Al-Sharif, (King Abdulaziz University, Faculty of Science & Arts) and a voucher specimen (no. CS-2-2020) was kept in the Faculty of Pharmacy`s herbarium.

Extraction and isolation

The dried rhizomes (7.0 kg) were grinded and extracted with $CHCl_3$ (6 times, 20 L each). The $CHCl_3$ extract was separated from the marc using sterile gauze (cotton) and then filtered through No. 1 Whatman filter paper. Under reduced pressure, the solvent was removed to yield a viscous brown residue (445 g) that was suspended in 200 mL distilled H_2O. Then, it was successively partitioned between *n*-hexane (8 × 2.0 L each) and chloroform (2.0 L × 8). Each fraction was concentrated to yield *n*-hexane (113.0 g) and chloroform (302.0 g) fractions. The *n*-hexane fraction was separated on SiO_2 column utilizing gradient EtOAc:*n*-hexane to afford mokko lactone, that was further purified using RP-18 column (H_2O:MeOH gradient) to produce mokko lactone as a yellow crystals.

Spectral data of mokko lactone

Yellow crystals; ESIMS *m*/*z*: 233 $[M+H]^+$; ^1H NMR ($CDCl_3$, 600 MHz): δ_H 5.20 (H-15A), 5.05 (H-15B), 4.88 (H-14A), 4.78 (H-14B), 3.92 (H-6), 2.88 (H-1), 2.81 (H-5), 2.54 (H-3, 8), 2.48 (H-9A), 2.11 (H-9B), 2.21 (H-11), 1.97 (H-7), 1.92 (H-2A), 1.83 (H-

2B), 1.23 (H-13); ^{13}C NMR ($CDCl_3$, 150 MHz): δ_C 178.7 (C-12), 151.2 (C-4, 10), 107.8 (C-15), 111.9 (C-14), 85.3 (C-6), 52.0 (C-5), 49.9 (7), 47.1 (C-1), 42.1 (C-11), 37.7 (C-9), 32.5 (C-3, 8), 30.2 (C-2), 13.2 (C-13).

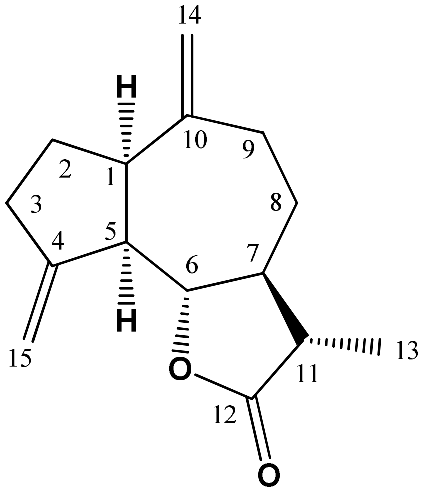

Figure S1: Chemical structure of mokko lactone.

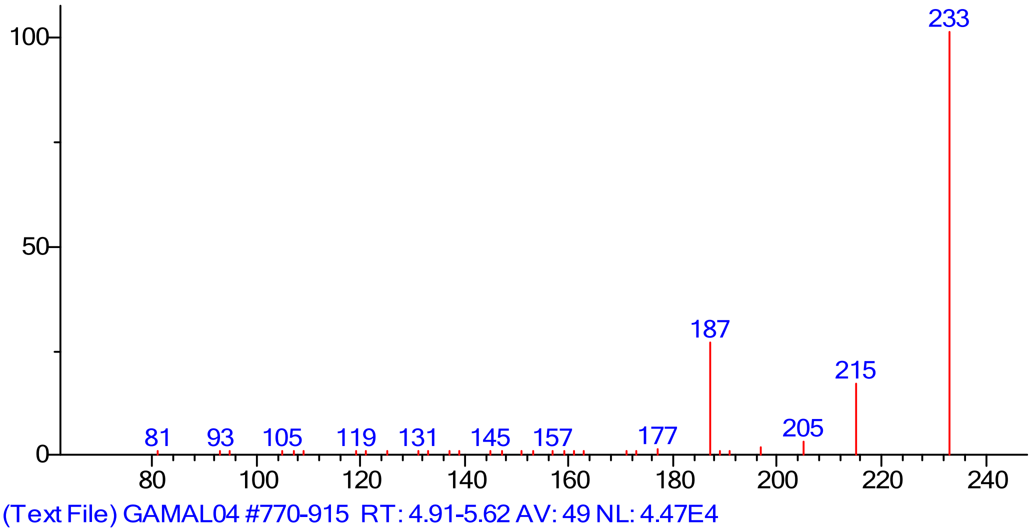

Figure S2: ESIMS of mokko lactone.

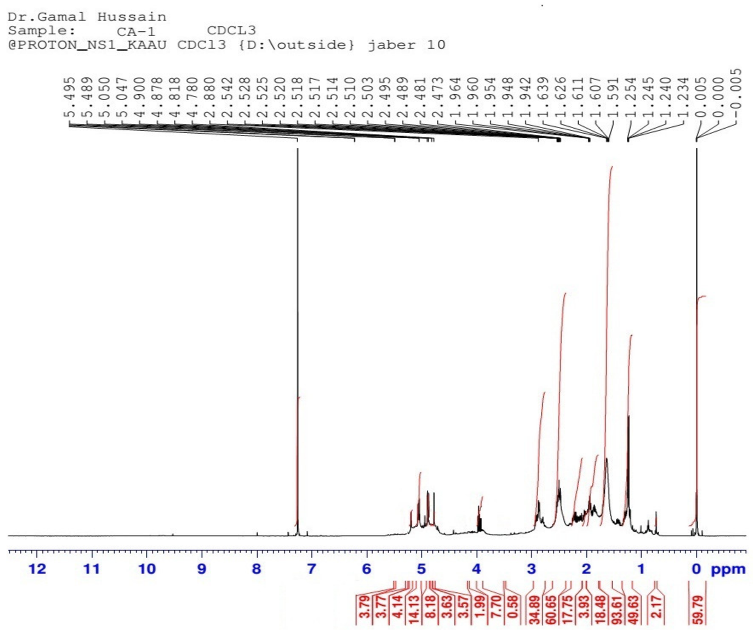

Figure S3: ^{1}H NMR spectrum of mokko lactone in $CDCl_3$ (600 MHz).

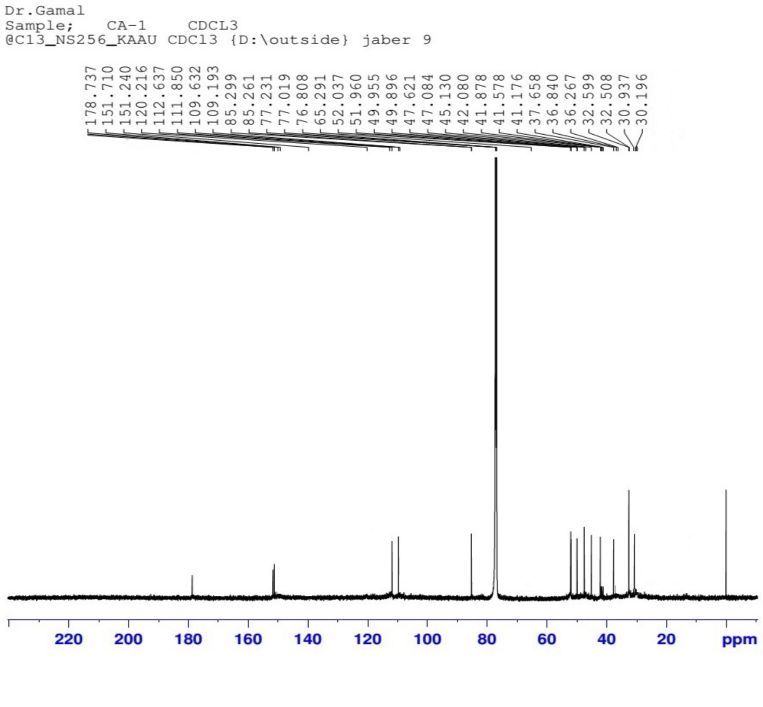

Figure S4: ^{13}C NMR spectrum of mokko lactone in $CDCl_3$ (150 MHz).
